# 3D-printed navigation templates combined with multimodal imaging assist low-grade glioma surgery: a retrospective cohort study

**DOI:** 10.3389/fsurg.2026.1687769

**Published:** 2026-03-13

**Authors:** Jun Qiu, Xin-Yi Cao, Yong Yi, Qing-Shan Deng, Jing-Cheng Jiang, Han Wang, Chao-Gui Zhang, Xu-Jun Shu

**Affiliations:** 1 Department of Critical Care Medicine, The Second People’s Hospital of Yibin, Yibin, Sichuan, China; 2 Department of Neurosurgery, The Second People’s Hospital of Yibin, Yibin, Sichuan, China; 3 Department of Neurosurgery, Jinling Hospital, Nanjing, China; 4 Department of Neurosurgery, Affiliated Jinling Hospital, Medical School of Nanjing University, Nanjing, China

**Keywords:** 3D reconstruction, 3D-printed templates, low-grade glioma (LGG), multimodal imaging, neuronavigation, surgical assistance

## Abstract

**Objective:**

To evaluate the feasibility of using patient-specific 3D-printed navigation templates combined with multimodal imaging for low-grade glioma (LGG) surgery.

**Methods:**

A retrospective cohort study was conducted involving 55 patients with supratentorial LGGs. Twenty-seven patients underwent surgery guided by a 3D-printed navigation template with multimodal 3D imaging (3D-printed group), and 28 received conventional neuronavigation-assisted surgery (control group). Perioperative outcomes—including operative time, tumor resection extent, intraoperative navigation usage, complications, and functional recovery—were compared.

**Results:**

The 3D-printed group had significantly shorter operative times (256.2 ± 9.8 vs. 271.6 ± 8.9 min, *P* < 0.05) and required fewer intraoperative navigation checks (0.2 ± 0.4 vs. 2.3 ± 1.6, *P* < 0.05). Tumor resection extent was comparable; residual volumes were categorized objectively (<10, 10–20, >20 cm³), with a trend toward lower residuals in the 3D group. Postoperative KPS scores and complication rates were similar between groups.

**Conclusion:**

Combining 3D-printed navigation templates with multimodal imaging enhances surgical precision and efficiency in LGG resection while reducing dependence on costly intraoperative systems. This approach is safe, cost-effective, and especially useful in resource-limited environments.

## Introduction

Low-grade gliomas (LGGs) present distinct surgical challenges. Despite technological advances, two persistent obstacles remain: precise tumor localization and intraoperative boundary delineation (1). Even with modern imaging, distinguishing diffuse LGG tissue from normal brain parenchyma remains difficult (2), and intraoperative brain shift—caused by edema, tumor removal, or cerebrospinal fluid loss—can degrade neuronavigation accuracy (3, 4), compromising surgical safety and resection completeness (5–7).

Conventional optical neuronavigation assists with preoperative planning and intraoperative guidance but has notable limitations. These systems require costly equipment and complex workflows, and their precision deteriorates during surgery due to brain deformation (8, 9). In resource-limited hospitals, economic and technical constraints often hinder their routine use (9, 10). Similarly, fluorescence-guided resection (e.g., 5-ALA) can enhance visualization but is restricted by high cost and availability (11). These challenges highlight the need for a more accessible, cost-effective alternative to support surgical accuracy in LGG treatment.

Recently, advances in three-dimensional (3D) imaging and 3D printing have introduced promising tools for neurosurgical planning (12). By integrating MRI, CT, and diffusion tensor imaging (DTI), multimodal 3D reconstruction can create patient-specific models that clarify the spatial relationships between tumors and adjacent eloquent structures (13–15). Building on this, 3D printing enables the fabrication of physical, patient-specific cranial templates for surgical guidance (16, 17). These guides allow accurate surface localization and craniotomy planning directly on the patient's skull (18–21). Once the digital infrastructure is in place, 3D printing becomes a relatively low-cost, reusable solution—particularly suitable for centers lacking advanced intraoperative imaging (22, 23).

In this study, we present the first clinical integration of 3D-printed navigation templates with multimodal 3D imaging for LGG surgery. Rather than aiming solely at time efficiency, our objective was to assess whether this technique could support accurate, safe, and practical tumor resection—with minimal reliance on real-time intraoperative technologies. We conducted a retrospective cohort analysis comparing key surgical outcomes—operative time, navigation usage, extent of resection, and functional recovery—between patients treated with the 3D-assisted approach and those undergoing standard neuronavigation. We also aimed to explore the technique's potential in training and in resource-constrained environments, where conventional intraoperative tools may be unavailable.

## Materials and methods

### Clinical data

We retrospectively analyzed 55 adult patients (18–75 years) with primary supratentorial low-grade gliomas (LGGs) treated at the Second People's Hospital of Yibin from January 2018 to October 2024. All surgeries were performed by the same senior neurosurgeon. Patients were assigned to either the 3D-printed group (*n* = 27) or the conventional group (*n* = 28). The allocation was non-randomized and partially influenced by the temporal implementation of the 3D-printing workflow; the conventional group primarily consisted of patients treated in the earlier phase of the study period, while the 3D-printed group comprised consecutive patients treated after the workflow was established. To minimize operator bias related to the learning curve, all surgeries were performed by the same senior neurosurgeon with over 15 years of experience in glioma surgery.Inclusion criteria comprised: (1) MRI/CT-confirmed single supratentorial lesion with postoperative histopathological diagnosis of LGG; (2) use of either 3D-printed or conventional neuronavigation guidance; (3) availability of complete clinical and imaging data. Exclusion criteria included: multiple lesions, systemic disease contraindicating surgery, prior oncologic treatment, severe psychiatric disorders, or loss to follow-up. Ethical approval was granted by the Institutional Ethics Committee (Approval No. 2023-161-01); informed consent was waived due to the retrospective nature of the study.

### Preoperative imaging and 3D reconstruction

All patients underwent high-resolution MRI (including contrast-enhanced 3D T1, T2-FLAIR, and DTI) and 256-slice CT (non-contrast, CTA, CTV). Imaging data were exported in DICOM format and co-registered using MATLAB R2018a and SPM12, with CT as the reference space. Segmentation and 3D model construction were performed in 3D Slicer (v4.10.1), integrating the following components: skull/skin surface (from CT), tumor (from T1 or T2-FLAIR), vasculature (from CTA/CTV), cortical surface (from CAT12), and subcortical tracts (from DTI). These elements were fused into a multimodal 3D model showing spatial relationships between the tumor and adjacent critical anatomy, aiding precise surgical planning.

### Preoperative planning and guide design

**Figure 1:**
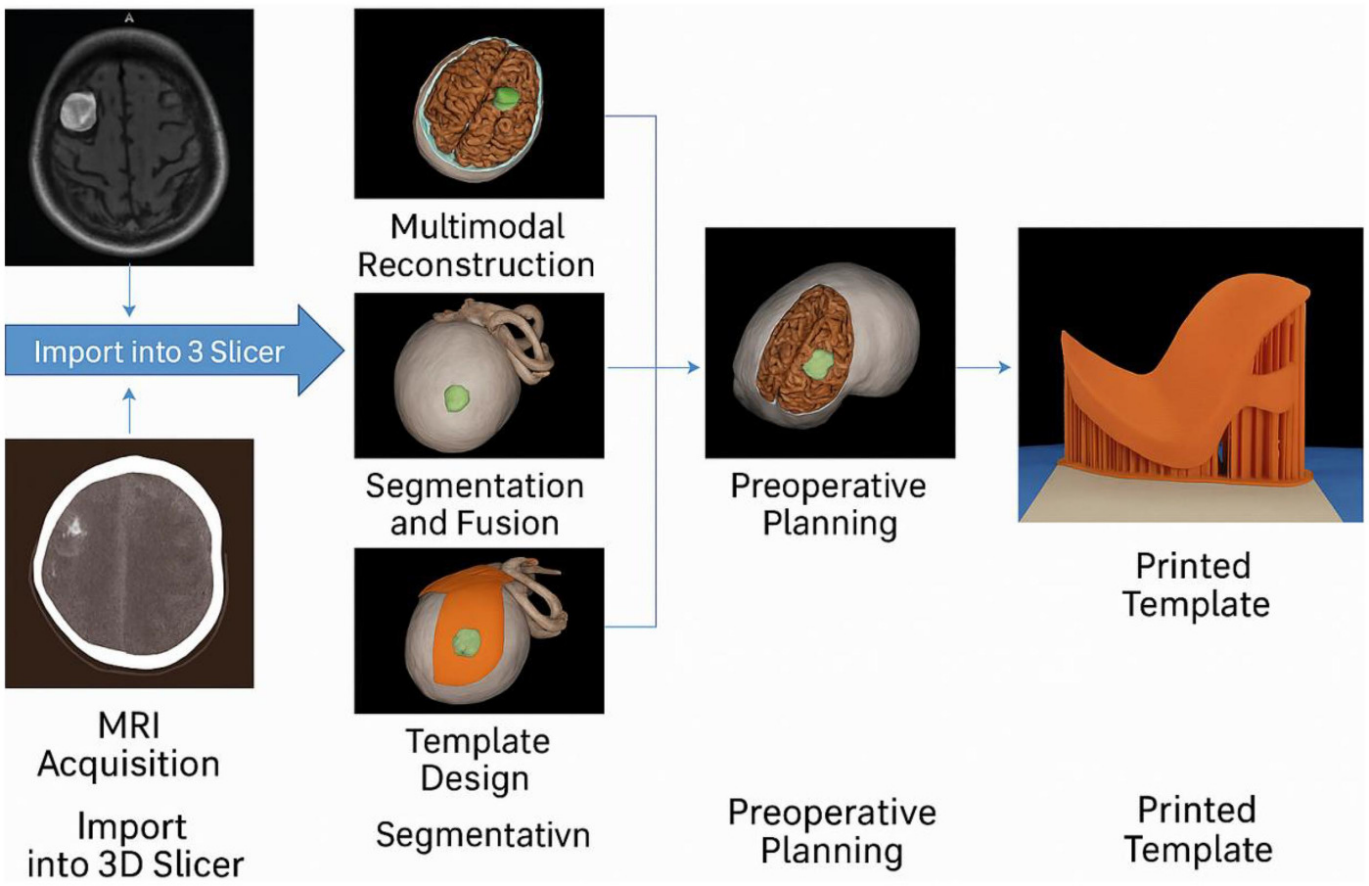
Workflow of multimodal image reconstruction and 3D-printed guide design. This figure outlines the stepwise process from MRI and CT acquisition to the final printed navigation template. Imaging data are imported into 3D Slicer for multimodal reconstruction, including segmentation and fusion of anatomical structures. A personalized guide is then designed and printed for precise intraoperative localization.

In the 3D group, the multimodal model was reviewed to define craniotomy margins, incision trajectory, and functional avoidance zones. Based on tumor surface projection, a patient-specific cranial guide was designed using 3D Slicer. The guide's contour matched cranial landmarks (e.g., supraorbital ridge, temporal line) to ensure stable positioning. The template outlined skin incision and craniotomy borders. STL files were exported and printed using PLA material on a desktop 3D printer. Each guide took ∼3 h to print and was sterilized with low-temperature ethylene oxide gas. The final product was a thin template for scalp/skull localization—not a 3D anatomical model (Figure 1).

### Surgical procedure

All patients underwent standard craniotomy and microsurgical resection under general anesthesia. In the 3D group, the printed guide was placed pre-incision for direct tumor projection localization. The preoperative 3D model was referenced intraoperatively to match cortical features, minimizing navigation use (kept on standby). Functional mapping was used as needed near eloquent cortex. In the conventional group, tumor localization, skin incision, and craniotomy were guided by optical neuronavigation based on preoperative MRI. No 3D printing or model fusion was used.

### Outcome measures

We compared the following perioperative outcomes:

1. Operative time: From localization (guide placement or navigation setup) to wound closure.
2. Tumor exposure: Postoperative overlay of craniotomy vs. tumor model to determine full or partial exposure.
3. Landmark concordance (3D group): Surgeons rated consistency between 3D model and intraoperative anatomy.
4. Navigation usage frequency: Number of times navigation was consulted during resection.
5. Residual tumor volume: Postoperative T2-FLAIR MRI (within 48–72 h), stratified as 0–10, 10–20, >20 cm³.Residual tumor volume was calculated based on postoperative T2-FLAIR MRI (acquired within 48–72 h). To ensure objectivity, volumetric measurements were performed by a senior neuroradiologist who was blinded to the surgical group allocation.
6. Postoperative complications: Rebleeding or infection within 30 days post-op.
7. Functional outcome: KPS at 3 months (categorized as >70 vs. ≤70).

### Statistical analysis

Analyses were performed in SPSS v22.0. Continuous data were compared using independent-sample t-tests (mean ± SD). Categorical and ordinal data (e.g., tumor volume, KPS) were analyzed using chi-square, Fisher's exact test, or rank-sum tests as appropriate. Statistical significance was defined as *P* < 0.05 (two-tailed).

## Results

### Baseline characteristics

A total of 55 patients (27 in the 3D-printed guide group, 28 in the conventional neuronavigation group) were included in the analysis. Baseline demographic and clinical characteristics were similar between the two groups (Table 1). There were no statistically significant differences in age (mean age ∼42 years in both groups), sex distribution, tumor volume, tumor location (left vs. right hemisphere), or preoperative physical status (ASA grade) (*P* > 0.5 for all comparisons). This indicates that the two groups were well-matched and comparable prior to intervention.

**Table 1:** Patient characteristics of the 3D-printed guide group vs. Conventional Navigation Group.

Characteristic	Conventional group (*n* = 28)	3D-printed group (*n* = 27)	t or *χ*²	*P* value
Age (years, mean ± SD)	42.3 ± 14.6	42.8 ± 18.1	0.112	0.911
Sex (Male: Female)	15: 13	16: 11	0.181	0.671
Tumor Volume (cm³)	72.7 ± 42.4	70.1 ± 39.1	0.232	0.817
Tumor Side (Left: Right)	13: 15	15: 12	0.458	0.498
ASA physical status I/II/III	14/11/3	15/9/3	Z = –0.338	0.735

No statistically significant differences were observed in baseline characteristics between the two groups. ASA, American society of anesthesiologists. Diagnoses were based on histological examination. Due to the retrospective nature of the study and resource constraints, complete molecular profiling (IDH mutation, 1p/19q codeletion) according to the WHO 2021 classification was not available for the entire cohort.

### Operative time

**Figure 2:**
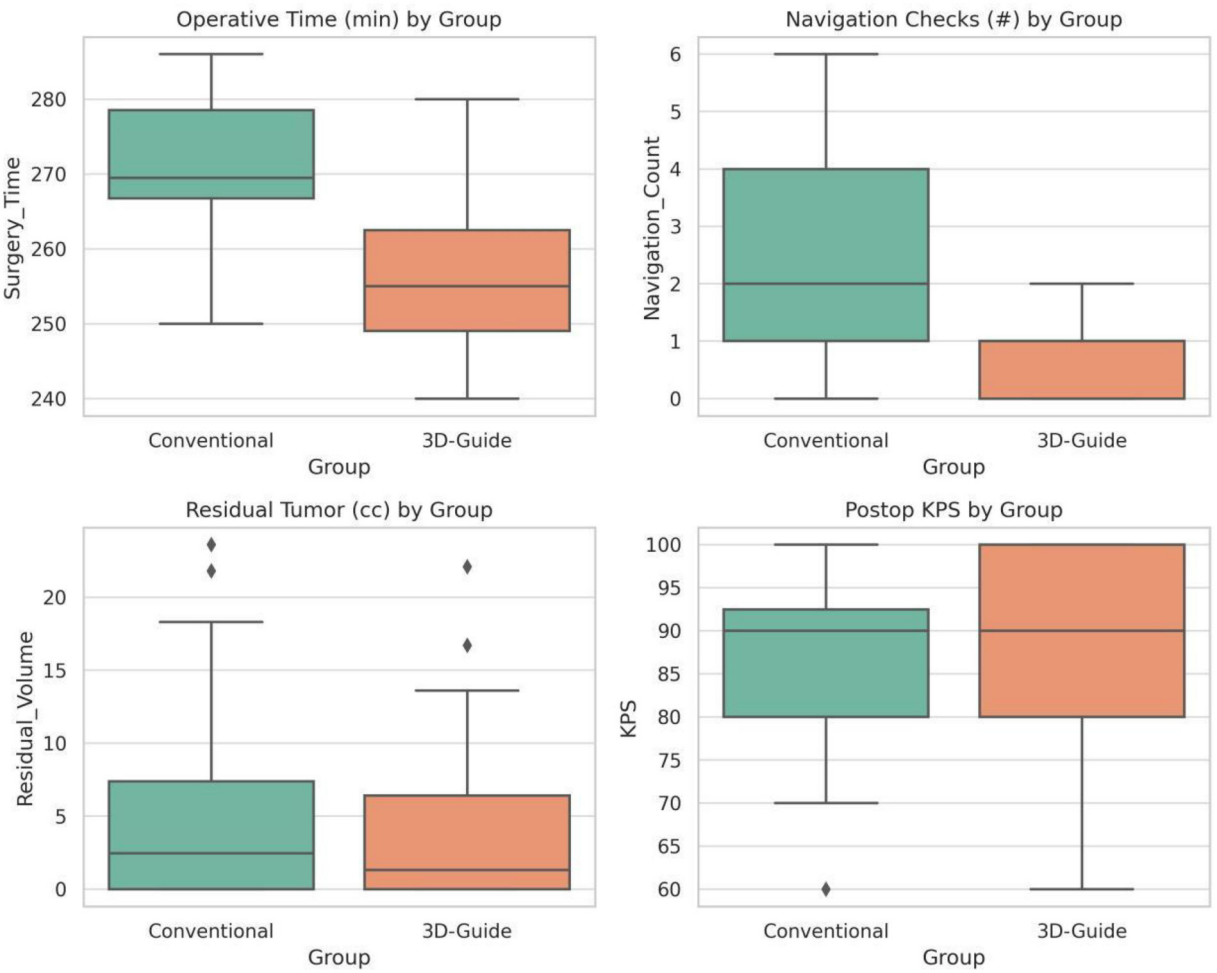
Box-plot comparison of key surgical parameters between groups. Top left: operative duration; top right: intraoperative navigation frequency; bottom left: postoperative residual tumor volume; bottom right: 3-month KPS. Each box shows the interquartile range (IQR) with the horizontal line as the median; the red line indicates the mean; whiskers represent 1.5× IQR; circles denote outliers. The 3D-printed guide group shows shorter operative times and fewer navigation checks, whereas residual tumor volume and postoperative functional outcomes display similar distributions across groups.

Using the 3D-printed navigation template significantly reduced the duration of surgery. The mean operative time in the conventional group was 271.6 ± 8.9 min, whereas in the 3D-printed group it was 256.2 ± 9.8 min. This corresponds to an average reduction of approximately 15 min when the 3D-printed guide was employed. Statistical analysis confirmed that the 3D-printed group's surgeries were significantly shorter on average than those in the conventional group (*P* < 0.001) (Figure 2). Detailed values are provided in Table 2. This improvement in operative efficiency is attributed to more rapid and confident localization of the tumor and planning of the craniotomy using the patient-specific guide, thereby streamlining the initial phases of surgery.

**Table 2:** Comparison of operative time, tumor exposure, and navigation usage.

Outcome measure	Conventional group	3D-printed group	Test statistic	*P* value
Operative duration (min)	271.6 ± 8.9	256.2 ± 9.8	t = 6.148	<0.001
Tumor exposure—complete (n)	23	25	*χ*² = 0.574	0.449
Tumor exposure—partial (*n*)	5	2		
Navigation usage (times per case)	2.32 ± 1.6	0.22 ± 0.4	t = 6.844	<0.001

### Intraoperative tumor exposure

Both surgical approaches achieved adequate exposure of the tumors in the vast majority of cases (Table 2). In the conventional group, 23 of 28 patients (82.1%) had complete tumor exposure through the craniotomy, while 5 patients had partial exposure (requiring retraction or extension of the bone window to fully access the tumor edges). In the 3D-printed guide group, 25 of 27 patients (92.6%) had complete exposure of the tumor on the initial opening, and only 2 patients had partial exposure. Although the 3D-guided group had a slightly higher complete exposure rate, this difference did not reach statistical significance (*P* > 0.05). In other words, both methods were generally effective in providing sufficient surgical access to the tumors, but the tailored guide ensured that nearly all tumors were directly underneath the pre-planned craniotomy.

### Anatomical landmark concordance

In the 3D-printed guide group, we assessed how accurately the preoperative 3D reconstructions predicted the surgical anatomy encountered. We found excellent concordance: in 24 out of 27 cases (88.9%), the key surface and subsurface anatomical features (such as the position of cortical gyri/sulci and major blood vessels relative to the tumor) seen during surgery were fully consistent with the multimodal 3D model prepared beforehand. In 2 patients (7.4%), the model was still helpful as a reference (partially consistent, aiding orientation), and only 1 case (3.7%) was rated as not useful because intraoperative anatomy did not match the model well. This high rate of anatomical accuracy demonstrates the reliability of the multimodal 3D reconstruction in guiding the surgeon. By contrast, in the conventional group no equivalent patient-specific model was used, so an objective concordance measure is not applicable; surgeons in that group relied on intraoperative navigation and their interpretation of standard MRI/CT images for anatomical guidance.

### Intraoperative navigation usage

**Figure 3:**
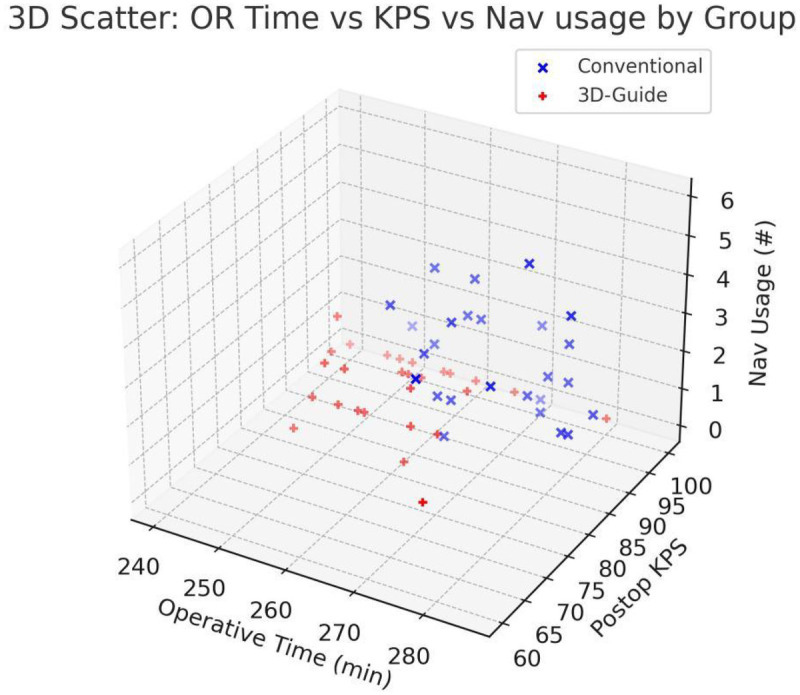
Three-dimensional scatter plot of operative time, 3-month KPS, and intraoperative navigation frequency. Patients in the conventional navigation group (blue “×”) cluster at longer operative times with more frequent navigation; patients in the 3D-printed guide group (red “+”) cluster at shorter operative durations with minimal navigation. Postoperative KPS was largely comparable between groups (mostly 80–100), indicating improved surgical efficiency with the 3D-printed guide without compromising neurological outcomes.

There was a notable difference in the reliance on real-time neuronavigation between the two groups (Table 2). In the conventional group, the navigation system was used frequently throughout the procedure, with an average of 2.32 ± 1.6 distinct navigational reference checks per surgery (aside from initial positioning). In the 3D-printed guide group, navigation was rarely needed: the average use was only 0.22 ± 0.4 times per case. In fact, most surgeries in the 3D guide group were completed with no intraoperative navigation at all beyond the initial patient registration. The difference in navigation usage frequency was statistically significant (*P* < 0.001). This indicates that the surgeons felt the patient-specific guide and the preoperative 3D visualization provided sufficient anatomical orientation such that continuous neuronavigation guidance was largely unnecessary in the 3D-assisted cases. Reducing dependence on neuronavigation can simplify the workflow and is especially advantageous in operating environments where such technology is not readily available or if navigation accuracy degrades over time due to brain shift (Figure 3).

### Residual tumor volume

Postoperative MRI scans were reviewed to quantify any residual tumor. Overall, both approaches achieved high rates of gross-total or near-total resection, with no statistically significant difference in residual tumor volume distribution between the two groups (Table 3). In the conventional group, 21 of 28 patients (75%) had minimal residual tumor (≤10 cm^3^), 5 patients (18%) had moderate residual (10–20 cm^3^), and 2 patients (7%) had > 20 cm^3^ of tumor remaining. In the 3D-printed group, 24 of 27 patients (89%) had ≤ 10 cm^3^ residual, 2 patients (7%) had 10–20 cm^3^, and 1 patient (∼4%) had > 20 cm^3^ residual. No statistically significant difference was found in this distribution (*P* > 0.4). Notably, the 3D-guided group had fewer patients with larger residual volumes, suggesting a trend toward more complete tumor removal, but the sample size was too small for this trend to reach significance. These results indicate that use of the 3D-printed template did not compromise the extent of resection—if anything, it showed a potential to modestly improve it, an aspect that would require further study with a larger cohort to verify.

**Table 3:** Comparison of postoperative tumor residuals, complications, and 3-month postoperative KPS scores between the Two groups.

Outcome	Conventional group (*n* = 28)	3D-printed group (*n* = 27)	Statistic	*P* value
Residual tumor volume—0–10 cm^3^	21	24	*χ*² = 1.810	0.404
Residual tumor volume—10–20 cm^3^	5	2		
Residual tumor volume— > 20 cm^3^	2	1		
Postoperative rebleed (yes: no)	2: 26	0: 27	–	0.491
Intracranial infection (yes: no)	2: 26	1: 26	–	1.000
3-month KPS ≤ 70 (yes: no)	2: 26	1: 26	–	0.736

### Postoperative complications and functional outcomes

Major complications were infrequent in both groups. In the conventional group, postoperative rebleeding and intracranial infection each occurred in 2 of 28 patients (7.1%). In the 3D-printed group, no rebleeding was observed, and one patient (3.7%) developed an infection. These differences were not statistically significant (both *P* > 0.4; Table 3).

**Figure 4:**
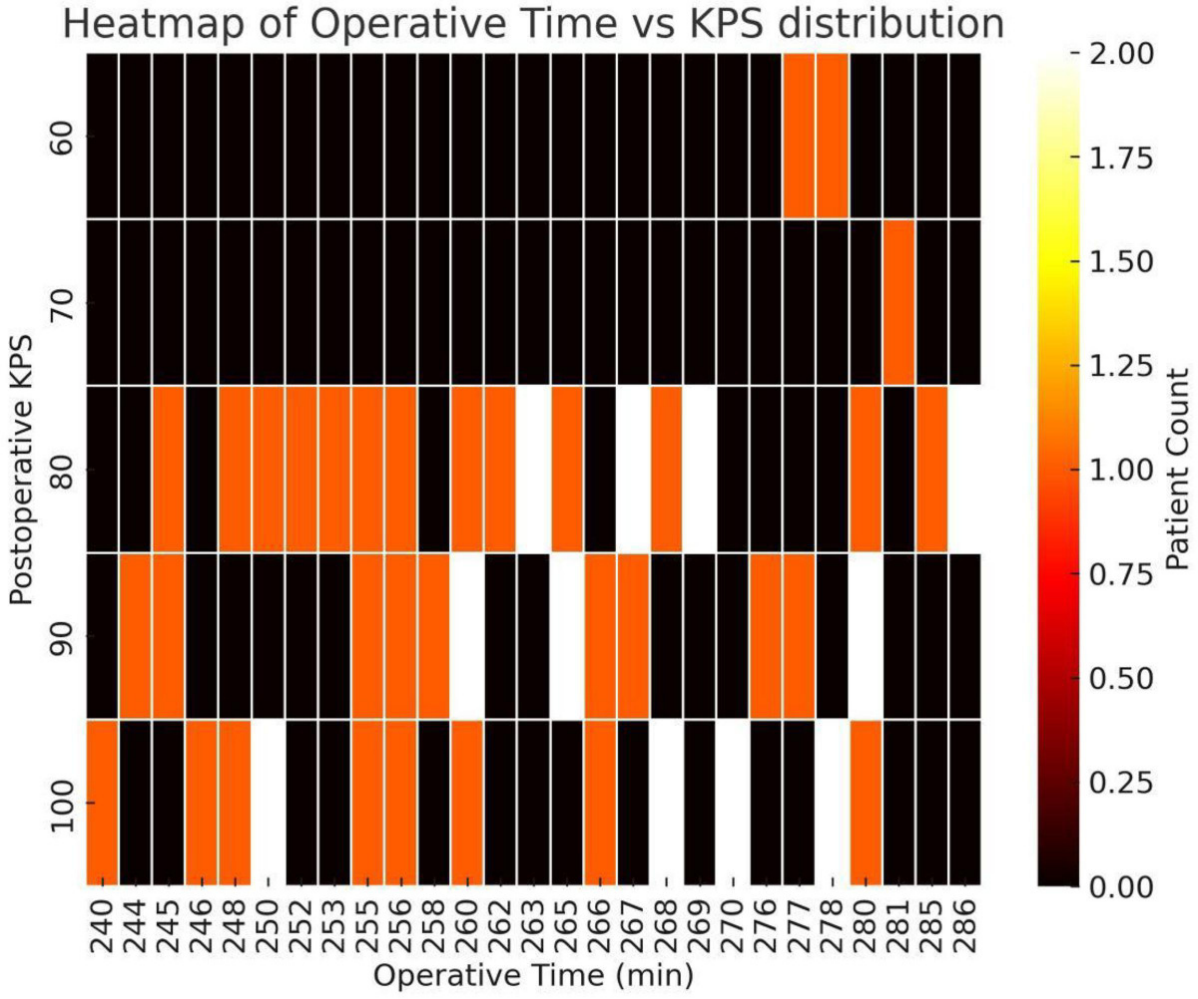
Heatmap of operative time vs. 3-month KPS (*n* = 55). *X*-axis: operative time (minutes); *Y*-axis: KPS at 3 months. Data are binned; color intensity reflects patient density (brighter = higher; black = 0). Most patients cluster at KPS 80–90 with operative time 250–270 min; KPS 100 cases also predominantly fall within 250–270 min. Operative times >280 min more often coincide with KPS <80. This pattern suggests a possible association between longer operations and poorer functional recovery, although it was not statistically significant in this cohort (*P* = 0.076).

Functional outcomes at 3 months were similarly favorable. The proportion of patients with KPS > 70 was 93% in the conventional group and 96% in the 3D group, with no significant difference in KPS distribution (*P* = 0.736; Figure 4). These results indicate that the 3D planning and guidance workflow did not compromise postoperative neurological function and is as safe as conventional neuronavigation.

We also explored the relationship between operative time and functional outcome. A heatmap analysis suggested a trend: patients with longer operative times (>280 min) were more likely to have lower KPS scores (<80) at 3 months. However, this correlation did not reach statistical significance (*P* = 0.076). While inconclusive, this trend implies that reducing operative time may contribute to better recovery—potentially reflecting lower surgical stress or anesthetic burden.

### Illustrative case

**Figure 5:**
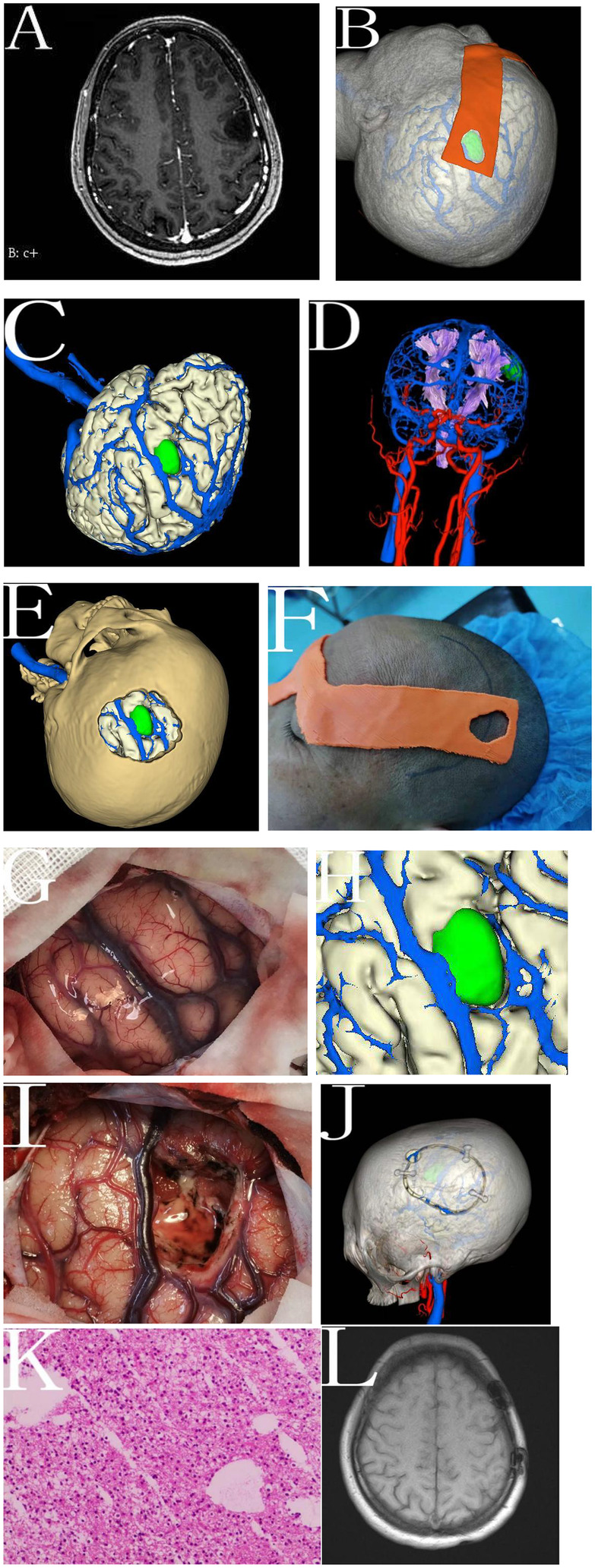
Multimodal 3D reconstruction and 3D-printed navigation template for an LGG in the left frontal lobe (illustrative case). **(A)** Preoperative enhanced MRI of the head showing a non-enhancing lesion in the left frontal lobe. **(B)** Preoperative design of the personalized 3D-printed navigation template. **(C)** Preoperative multimodal imaging 3D reconstruction showing the tumor located in the lower part of the precentral gyrus. **(D)** The tumor compressing surrounding fiber tracts. **(E)** Preoperative bone flap design. **(F)** Intraoperative use of the personalized navigation template for localization. **(G)** Intraoperative exposure of brain tissue after dural opening. **(H)** Comparison of the intraoperative findings with the enlarged preoperative 3D reconstructed images to determine the resection range. **(I)** Complete resection of the tumor. **(J)** Postoperative 3D reconstructed images verifying the position of the tumor relative to the bone window. **(K)** Postoperative pathological results showing astrocytoma (IDH mutant, WHO grade II, H&E staining, 10×). **(L)** MRI of the head three months postoperatively showing complete tumor resection with a residual volume of 0 cm^3^.

A 48-year-old woman with a left frontal non-enhancing lesion (∼1.7 × 1.8 × 2.1 cm) suspected as low-grade glioma underwent resection using a patient-specific 3D-printed guide. Multimodal 3D reconstruction localized the tumor near the precentral gyrus and motor tracts, guiding the design of a skull-conforming template based on anatomical landmarks. Intraoperatively, the guide enabled accurate craniotomy placement without neuronavigation. Cortical anatomy aligned well with the preoperative model, and mapping confirmed the lesion's location anterior to motor cortex. The tumor was resected under microscopy using the printed guide and anatomical cues alone. Postoperatively, the patient had no neurological deficits, and MRI confirmed gross total resection. At 3 months, her KPS score was 90 with no recurrence (Figure 5).

## Discussion

This study evaluated a novel workflow integrating patient-specific 3D-printed cranial navigation templates with multimodal imaging-based 3D reconstruction for low-grade glioma (LGG) surgery (24). The approach significantly shortened operative preparation time, reduced dependence on intraoperative neuronavigation (25), and maintained similar safety and efficacy compared with conventional neuronavigation techniques (26, 27). These findings suggest that the method is a safe, feasible, and cost-effective adjunct, particularly beneficial in resource-limited settings where advanced intraoperative technologies may be unavailable (28).

The 3D-printed guides demonstrated precision and practicality, enabling accurate cortical target localization by translating digital models into patient-specific physical templates (29, 30). This “pre-navigation” step streamlined skin-incision planning and craniotomy design, resulting in fewer neuronavigation checks and a lower cognitive workload. Although the absolute reduction in operative time (∼15 min) represents a modest fraction of the total surgical duration, its clinical significance lies in the phase where it occurs. This time saving was achieved primarily during pre-incision localization and craniotomy planning—a high-cognitive-load period for the surgical team. By streamlining this initial setup, the 3D template reduces the cumulative anesthesia duration and minimizes the “setup fatigue” associated with complex navigation registration. In high-volume or resource-constrained centers, such efficiency gains, when aggregated across multiple cases, can meaningfully improve operating room turnover and resource utilization (31). Overall, this clinically feasible workflow, compared with conventional neuronavigation, was associated with a simpler setup, a shorter operative time, and lower costs (32, 33).

Multimodal 3D reconstruction was central to the workflow. By integrating MRI, CT, and DTI data, a comprehensive spatial map of tumor and adjacent eloquent anatomy was generated. The model furnished reliable preoperative spatial orientation, achieving complete intraoperative–anatomical concordance in ∼89% of cases (34).For LGGs with ill-defined borders, especially near functional cortices, such preoperative planning served as a mental “GPS”, enhancing both safety and extent of resection (35).

A notable outcome was the reduced reliance on intraoperative navigation. Surgeons in the 3D group often proceeded confidently with resection after guide-assisted exposure, requiring significantly fewer navigation checks than controls. This reduced disruption, cost, and reliance on systems potentially compromised by brain shift. The “navigation-light” model—combining physical templates and preoperative mental mapping—may represent a pragmatic alternative in many surgical contexts (36, 37).

The quality of tumor resection was comparable between groups. Although differences were not statistically significant, the 3D group demonstrated a lower proportion of cases with residual tumor volumes >10 cm^3^ and no cases exceeding 20 cm^3^. To facilitate objective evaluation, residual volumes were categorized as 0–10 cm^3^, 10–20 cm^3^, and >20 cm^3^, following established thresholds in prior studies (38, 39). Notably, the reduced operative time and decreased reliance on intraoperative navigation observed in the 3D group were not associated with higher complication rates. Functional recovery was similarly favorable in both groups, with over 90% of patients achieving a Karnofsky Performance Status (KPS) score >70 at 3 months (40, 41).

This technique is especially promising for centers lacking access to neuronavigation, fluorescence, or intraoperative MRI. While IOUS is available at our center, we found it suboptimal for LGG due to its limited contrast resolution. We therefore excluded it from this workflow. However, we acknowledge that 3D printing and IOUS could be complementary, with the former supporting initial localization and the latter aiding intraoperative reassessment. Future studies should explore this hybrid strategy, particularly in LGGs prone to significant brain shift (42, 43).

Beyond clinical use, the workflow offers educational value. 3D-printed guides can serve as tactile training tools for junior neurosurgeons to better understand cortical anatomy and surgical planning—especially where cadaveric training or advanced navigation is limited. As 3D printing becomes more accessible, this technique may help democratize neurosurgical precision globally.

Several limitations to this study should be considered. First, the retrospective, non-randomized design introduces potential temporal bias, as the conventional navigation group largely predated the routine use of the 3D-printing workflow. While general perioperative care may have evolved over the 6-year study period, the consistent involvement of a single highly experienced senior neurosurgeon helps mitigate the impact of a surgical learning curve. Second, the relatively small sample size (*n* = 55) limits the statistical power to detect differences in rare complications or subtle variations in the extent of resection. Consequently, the trend toward lower residual tumor volumes in the 3D-guided group should be interpreted cautiously as a demonstration of feasibility and non-inferiority, rather than definitive superiority. Third, owing to the retrospective timeframe and institutional resource constraints, full molecular classification (per WHO 2021 criteria) was not uniformly available, and diagnoses relied primarily on histological confirmation. Finally, focusing exclusively on supratentorial LGGs treated by a single surgeon limits the external validity of these findings.

Despite these limitations, this workflow was purpose-built to effectively reduce surgical setup burden. By relying on standard preoperative imaging and a sterilizable, patient-specific 3D-printed template, the technique avoids dependence on expensive, proprietary navigation hardware and requires minimal initial registration. Compared with conventional neuronavigation, this model-driven approach offers a simpler setup, fewer components, shorter preparation times, and lower overall expense. Particularly in resource-constrained settings, it provides dependable anatomical orientation and can serve as a pragmatic, cost-effective substitute when real-time neuronavigation is unavailable. Future prospective, multi-center studies are required to definitively validate its non-inferiority against standard navigation across diverse pathologies and less experienced surgical teams. Additionally, streamlining the preoperative pipeline with AI-assisted segmentation and augmented reality (AR) overlays could further reduce preparation time and shorten the learning curve (44, 45).

## Conclusion

This study shows that combining patient-specific 3D-printed cranial templates with multimodal 3D imaging is a practical and effective aid for low-grade glioma surgery. The approach enhances preoperative planning, supports precise tumor localization, and shortens operative time, while maintaining resection quality and safety comparable to conventional neuronavigation. As a low-cost, reproducible workflow—particularly valuable where access to advanced intraoperative tools is limited and in training environments—it translates detailed imaging into visual and tactile guidance and, with further validation, may serve as a useful complement to existing neurosurgical workflows.

## Data Availability

The raw data supporting the conclusions of this article will be made available by the authors, without undue reservation.
